# Hierarchically Engineered Flame‐Retardant Triboelectric Yarn Exhibiting Robust Mechanical Performance and Humidity‐Boosted Electrical Output for Firefighting Applications

**DOI:** 10.1002/advs.202507673

**Published:** 2025-08-12

**Authors:** Ying Sun, Yong Zhang, Haoyu Shi, Shujuan Wang, Jinlin Liu, Yu Luo, Cheng Zhang, Kun Zhang, Shi‐xiong Li, Wei Fan

**Affiliations:** ^1^ School of Textile Science and Engineering Key Laboratory of Functional Textile Material and Product of the Ministry of Education Xi'an Polytechnic University Xi'an Shaanxi 710048 China; ^2^ School of Chemistry Xi'an Jiaotong University Xi'an Shaanxi 710049 China; ^3^ College of Textiles Donghua University Shanghai 201620 China; ^4^ Shaanxi Yuanfeng Prosafe Co., Ltd. Xi'an Shaanxi 710025 China

**Keywords:** flame retardant performance, intelligent firefighting suits, self‐powered sensor, triboelectric nanogenerators

## Abstract

As urbanization progresses and building complexity grows, fire safety gains prominence, driving up demand for intelligent, high‐performance fire‐fighting gear. This study develops a super‐intelligent fireproof yarn (SIFY) with a coaxial structure via hierarchical textile assembly. The middle waterproof and flame‐retardant layer and the outer high‐strength flame‐retardant poly(p‐phenylene benzobisoxazole) fibers endow SIFY with top‐notch flame‐retardant performance (limiting oxygen index of 50.2%), high tensile strength (550.72 MPa), and remarkable wear resistance (over 12 000 rubbing cycles). These properties are currently the best among reported triboelectric yarns. Notably, the innovative design of SIFY ingeniously overcomes the limitations of triboelectric nanogenerators in humid environments. It achieves a remarkable breakthrough where the triboelectric performance increases rather than decreases with rising relative humidity. The unique properties endow SIFY with the capabilities to function as both a power source and a self‐powered sensor in intelligent firefighting supplies. It can be utilized in gesture‐recognition systems and intelligent firefighting ropes, thereby ensuring safer and more efficient fire‐fighting operations for firefighters.

## Graphical abstract

Inspired by a high‐temperature‐resistant wire, a Super Intelligent Fireproof Yarn is developed through the technology of hierarchical textile assembly. This yarn exhibits high tensile strength, shear resistance, and excellent flame‐retardant performance, and achieves humidity‐enhanced triboelectric output, enabling it to meet the demands of extreme scenarios like fire rescue.

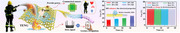

## 1 Introduction

At present, the development of intelligent firefighting suits primarily follows two directions. One approach involves sewing or affixing existing rigid sensors, such as temperature sensors, humidity sensors, and heart rate sensors, onto the current firefighting suits.^[^ ^1^, ^2^ ^]^ Nevertheless, this method has glaring drawbacks. It not only impairs the flexibility and breathability of the firefighting suits, thus limiting the mobility of firefighters, but also, since these sensors are powered by batteries, existing batteries carry risks of leakage and explosion in high‐temperature conditions, posing new safety threats to firefighters.^[^ ^3^, ^4^, ^5^ ^]^ The other direction is to develop a flexible, flame‐retardant, textile‐based self‐powered sensor.^[^ ^6^, ^7^, ^8^ ^]^ Cheng et al. developed a fabric‐based triboelectric nanogenerator (TENG) based on flame‐retardant cotton fabric. It has excellent flame‐retardancy. After burning continuously for 20 s, its output voltage decreased by only ≈6.5%.^[^ ^9^ ^]^ However, the strength of the cotton‐fabric‐based TENG cannot meet the high‐strength requirements of firefighting suits. Ma et al. fabricated a yarn‐based TENG with both high strength and flame‐retardant functions by winding polyimide yarns around conductive yarns. This TENG can accurately locate the position of survivors, providing great assistance to rescue work.^[^ ^10^ ^]^ However, all the currently reported textile‐based TENGs have a common issue: environmental humidity can cause a decrease in their electrical performance output, which in turn has a negative impact on signal transmission.^[^ ^11^, ^12^, ^13^ ^]^ Therefore, the development of textile‐based TENGs that possess high strength, excellent flame‐retardant properties, and resistance to humidity is essential for their application in fire‐fighting scenarios.

In this study, inspired by a widely used high‐temperature resistant wire (Figure S1, Supporting Information), we developed a mechanically robust, flame‐retardant, and humidity‐enhanced yarn‐based TENG for firefighting, named Super Intelligent Fireproof Yarn (SIFY). The commercial wire features a coaxial structure. The core metal wire conducts electricity, ensuring stable current transmission. The silica‐gel middle layer insulates the wire from moisture and other interference, guaranteeing its safe operation in complex environments. The outermost braided glass‐fiber sheath enhances the wire's overall strength, durability, and impact resistance, thus prolonging its service life. SIFY also adopts a coaxial structure achieved through hierarchical textile assembly. The core layer consists of a silver‐plated copper wire wound around Polydimethylsiloxane (PDMS), serving as a telescopic electrode that resists breakage under certain deformation. A coaxial wet‐spinning technique is used to coat the core yarn with a layer of styrene‐ethylene‐butylene‐styrene block copolymer (SEBS)/ammonium polyphosphate (APP)/phosphorus‐nitrogen flame retardants (PNFR) elastic material, which acts as the middle layer. This middle layer is moisture‐resistant and flame‐retardant, ensuring stable triboelectric output of SIFY. The outermost layer is made of Poly(p‐phenylene benzobisoxazole) (PBO) fibers applied via coaxial two‐dimensional braiding technology. These fibers offer high mechanical strength and flame retardancy, endowing SIFY with high tensile strength, shear resistance, and excellent flame‐retardant performance to meet the demands of extreme scenarios like fire rescue.

As a novel textile yarn, SIFY exhibits outstanding textile processing performance. It can not only be applied independently but also seamlessly integrate with various commercial textile fibers, giving rise to a variety of electronic fabrics. These fabrics combine the softness of traditional fabrics with the functionality of electronic components. In this study, SIFY was knitted into an electronic fabric with certain deformability, which can be used as a power source, a self‐powered sensor, and a strain sensor. Moreover, SIFY braided with commercial aramid fibers forms an intelligent fire‐fighting rope with online health‐monitoring functions, providing a guarantee for the operational safety of firefighters.

## 2 Results and Discussion

### 2.1 Structure and Properties of SIFY

**Figure 1:**
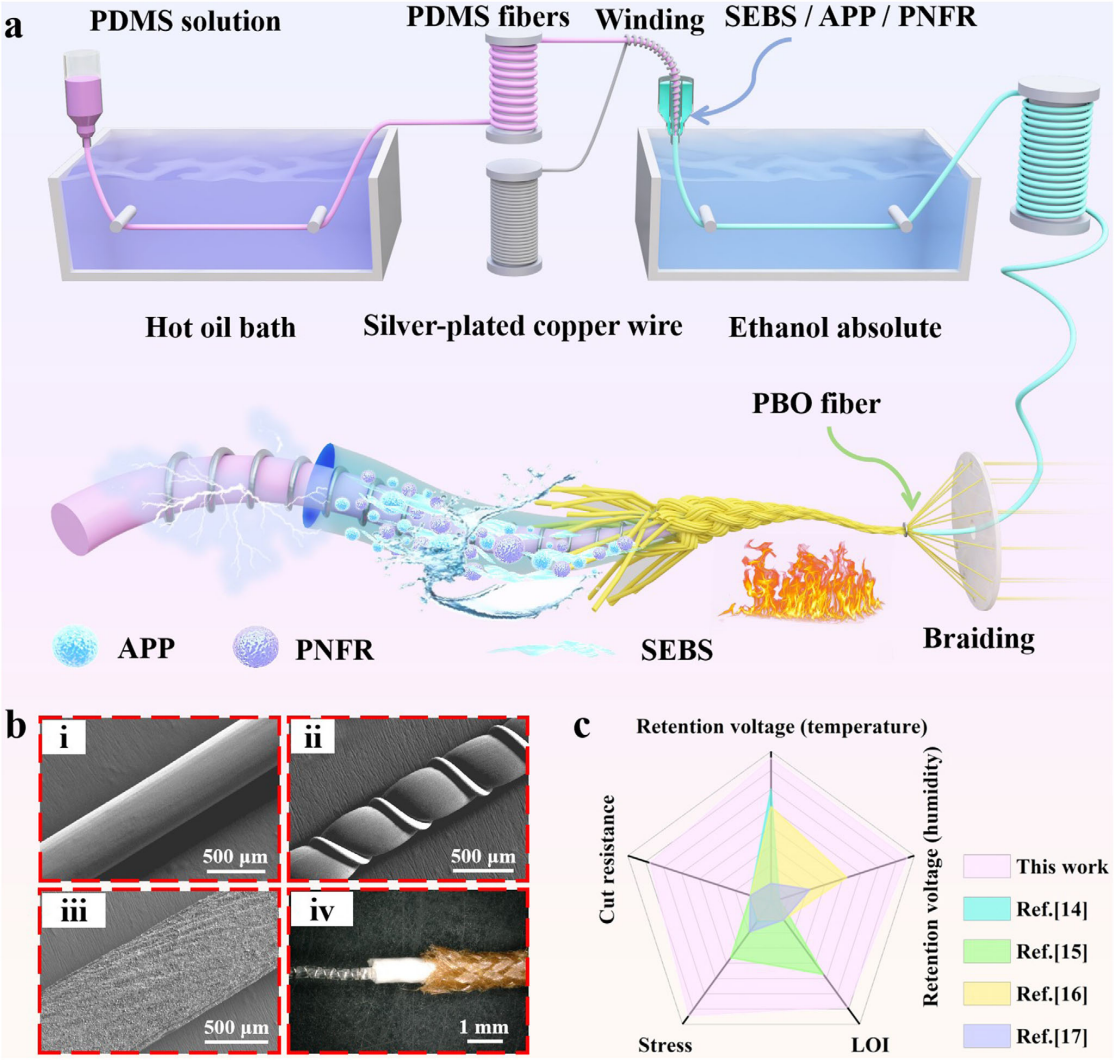
Preparation process, morphology, and properties of super intelligent fireproof yarn (SIFY). a) SIFY preparation flowchart. b) SEM and super‐depth‐of‐field images of SIFY: i. PDMS fiber; ii. PDMS/Silver‐plated copper wire; iii. Flame‐retardant and waterproof triboelectric yarn (FWY); iv. SIFY. c) A comparison chart of the key core indicators between SIFY and the currently reported flame‐retardant triboelectric nanogenerator yarns.

**Figure** **1**a,b present the preparation and structural schematic of SIFY. The PDMS fiber has a uniform diameter and smooth surface, prepared by wet spinning. (Figure 1b–i). A highly conductive (1.92 × 10^8^ S m^−1^) silver‐plated copper wire was evenly wrapped around the PDMS fiber to form the elastic conductive yarn (Figure 1b–ii). SEBS/APP/PNFR was uniformly coated on the outer surface of the PDMS/Silver‐plated copper wire via coaxial wet‐spinning to form the flame‐retardant and waterproof yarn (FWY), as shown in Figure 1b‐iii. PBO fibers were braided on the FWY to form SIFY with a uniform three‐layer coaxial structure (Figure 1b‐iv). The comparison of the mechanical properties of the above‐mentioned parts is shown in Figure S2 (Supporting Information). We conducted cut resistance tests on ultra‐high molecular weight polyethylene (UHMWPE), FWY, and SIFY fabrics, and SIFY has good cut resistance performance (Figure S3 and Video S1, Supporting Information).

Benefiting from the high electrical conductivity of the core layer, the excellent waterproof and flame‐retardant properties of the middle layer, and the superior mechanical and flame‐retardant properties of the outermost PBO fibers, SIFY outperforms all currently reported flame‐retardant nanogenerator yarns in terms of high‐temperature electrical retention rate, humidity electrical retention rate, limiting oxygen index (LOI), tensile stress, and cut resistance (Figure 1c).^[^ ^14^, ^15^, ^16^, ^17^ ^]^ The specific data are shown in Table S1 (Supporting Information).

### 2.2 Flame Retardancy of SIFY

**Figure 2:**
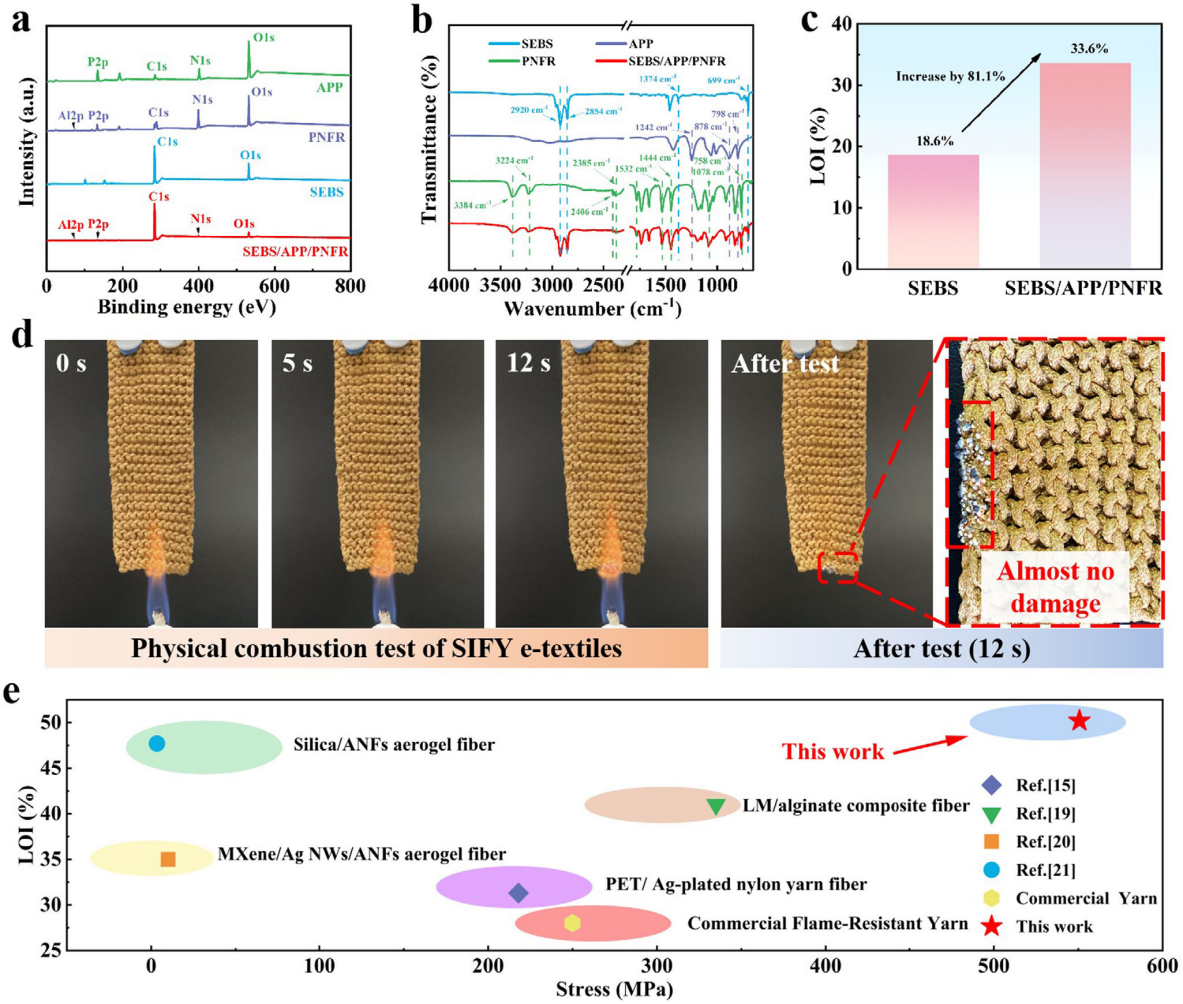
Flame‐retardant properties of SIFY. a) XPS analysis of SEBS, APP, PNFR, and the SEBS/APP/PNFR. b) Infrared spectrum of SEBS, APP, PNFR, and the SEBS/APP/PNFR. c) LOI of SEBS and SEBS/APP/PNFR. d) Physical burning diagram of SIFY e‐textile. e) Stress and LOI comparison between SIFY and other reported flame‐retardant yarn‐based TENGs.

SEBS is a thermoplastic elastomer with excellent flexibility, aging resistance, and processability.^[^ ^18^ ^]^ However, its molecular structure contains many carbon‐hydrogen bonds, making it prone to combustion when exposed to a fire source. During combustion, it releases heat and smoke and may even produce molten droplets, which can spread the fire. To address this issue, we added APP and PNFR flame retardants to the SEBS spinning solution to obtain the SEBS/APP/PNFR flame‐retardant material. The characteristic elements (Al, P, N) of PNFR and the characteristic elements (P, N) of APP are successfully observed in the SEBS/APP/PNFR (**Figure** **2**a), which proves that APP and PNFR have been successfully added to SEBS. However, the infrared spectral analysis of these materials reveals that they are only physically mixed without any chemical reactions occurring (Figure 2b).

This is because in the infrared spectrum of SEBS/APP/PNFR, apart from the characteristic peaks of APP and PNFR, no new characteristic peaks appear. Pure SEBS has no flame‐retardant property, which would turn into ashes under the flame directly. In contrast, after flame‐retardant modification, the flame‐retardant property of SEBS/APP/PNFR is significantly enhanced, and it achieves self‐extinguishing upon removal from the flame. As shown in Figure 2c, the LOI values of SEBS/APP/PNFR is up to 33.6%, which is 81.1% higher than that of SEBS (18.6%). In addition, SEBS/APP/PNFR exhibits a continuous and dense carbon layer and bubbles on its surface (Figure S4, Supporting Information). The bubbles help to form a charred or expanded protective layer so that it can isolate oxygen and reduce its contact with combustibles, which enhances the flame‐retardant performance of SEBS/APP/PNFR.

The SIFY exhibits extremely excellent flame‐retardant performance, which is attributed to the outstanding flame‐retardant properties of the intermediate layer SEBS/APP/PNFR and the outermost layer of PBO fibers. Apparently, after undergoing a 12 s combustion test, the damaged length of the fabric is negligible (Figure 2d; Video S2, Supporting Information). In addition, the SIFY exhibits a LOI of 50.2% and a tensile strength of 550.72 MPa, surpassing the reported flame‐retardant yarn‐based TENGs (Figure 2e).^[^ ^15^, ^19^, ^20^, ^21^ ^]^ More importantly, compared with the aramid fiber yarns (LOI = 29%, tensile strength = 250 MPa) commonly used in fire‐fighting suits, the SIFY also performs better in these two aspects, laying the foundation for its application in intelligent fire‐fighting suits.

### 2.3 Triboelectric Properties of SIFY

**Figure 3:**
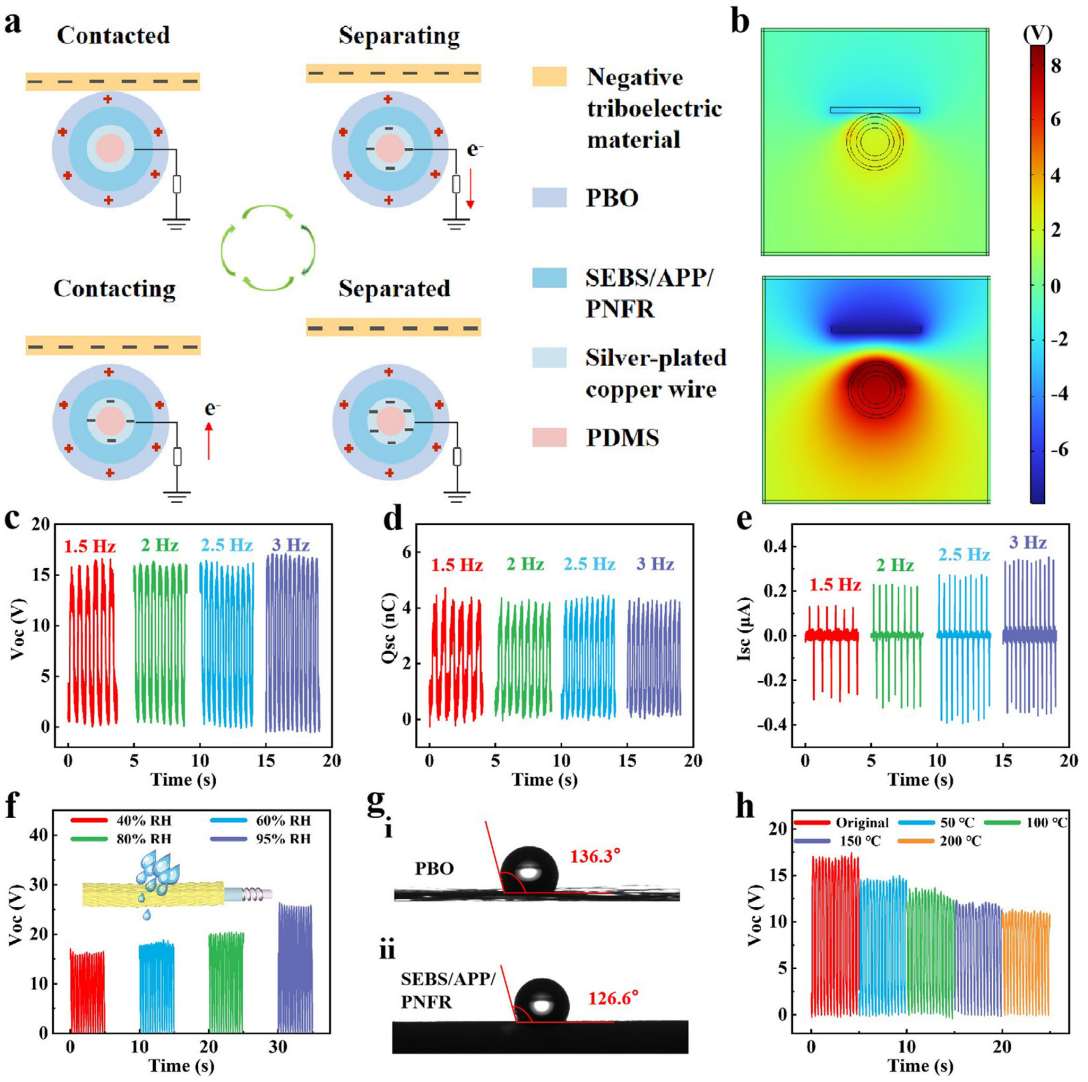
Triboelectric properties of SIFY. a) Working principle diagram of SIFY. b) The electrostatic potential distribution of SIFY simulated by COMSOL software. c–e) Open‐circuit voltage (V_OC_), short‐circuit charge transfer (Q_SC_), and short‐circuit current (I_SC_) of SIFY under different excitation frequencies (1.5–3 Hz). f) The V_OC_ of SIFY under different humidity conditions. g) The water contact angle of PBO fiber and SEBS/APP/PNFR. h) The V_OC_ of SIFY under different temperature conditions.

SIFY operates in the single‐electrode mode, and its working principle is illustrated in **Figure** **3**a. In this mode, the outermost layer of SIFY serves as the positive‐electrode friction material, the negative‐electrode friction material (such as polyurethane) is used as the contact object, and the silver‐plated copper wire is used as the conductive electrode. The SIFY yarn generates energy through the periodic contact and separation movements between the outermost layer of SIFY and the negative‐electrode friction material. The electrostatic potential distribution of SIFY during the contact and separation processes was simulated by COMSOL software, as shown in Figure 3b. Figure 3c–e displays the output performance of SIFY with a length of 3.5 cm under a 60 N force at different frequencies. When the contact frequency rises from 1.5 to 3 Hz, open‐circuit voltage (V_OC_) and short‐circuit charge transfer (Q_SC_) of SIFY stay relatively stable at 16.8 V and 4.1 nC, respectively (Figures 3c,d). Meanwhile, the short‐circuit current (I_SC_) of SIFY increases from 0.13 to 0.34 µA (Figure 3e). To investigate the relationship between output voltage and yarn length, SIFY samples of varying lengths were fabricated for electrical characterization. Results demonstrate an increasing trend in output voltage with length under consistent internal structure conditions (Figure S5, Supporting Information).

Most fires are extinguished by water. Consequently, firefighters are often in an environment with high relative humidity (RH), usually exceeding to 60%. Yet, most of textile fibers absorb moisture, generally diminishing the V_OC_ of textile‐based TENGs and impeding their application in firefighting.^[^ ^22^, ^23^ ^]^ In this work, the innovative design of SIFY ingeniously overcomes the shortcomings of TENGs in humid environments, achieving a breakthrough where the triboelectric performance increases rather than decreases with the increase of RH. As shown in Figure 3f, before RH reaches to 80%, the V_OC_ of SIFY shows a slow upward trend. However, when RH climbs to 95%, the V_OC_ value increases significantly, reaching 53.57% of its initial value, and then stabilizes in the range of 25–27 V. The main reason for this abnormal phenomenon is as follows: First, the middle‐layer SEBS/APP/PNFR is water‐repellent, effectively preventing external moisture from affecting the core‐layer electrode. Second, both the outer‐layer PBO fibers and SEBS/APP/PNFR are hydrophobic with weak moisture absorption. And the water contact angle is 136.3° and 126.6° for PBO fiber and SEBS/APP/PNFR, respectively, which is shown in Figure 3g. Thus, the V_OC_ of SIFY changes slightly below 80% RH. However, at 95% RH or higher, moisture diffuses along the PBO‐SEBS/APP/PNFR interface (Figure S6, Supporting Information) under capillary action. Moisture forms hydrogen bonds with PBO fibers, and water molecules participate in triboelectrification as more positively‐charged materials, leading to higher V_OC_.^[^ ^24^, ^25^ ^]^ To further illustrate the enhancement of triboelectric performance by moisture incorporation, we conducted COMSOL simulations (Figure S7, Supporting Information). The results confirm that the involvement of moisture improves triboelectric characteristics. Infrared spectroscopy further verifies the hydrogen‐bond formation (Figure S8, Supporting Information).

In fire rescue, besides the influence of moisture on triboelectricity, there is also the influence of high temperature.^[^ ^26^, ^27^, ^28^ ^]^ As shown in Figure 3h, when the temperature increases from room temperature to 200 °C, the output voltage of the TENG decreases. At room temperature, the V_OC_ of SIFY is 16.8 V. When the temperature reaches to 200 °C, the V_OC_ of SIFY remains at 66.57% of the original SIFY, indicating that SIFY can work effectively in a wide range of temperature environments. Notably, the electrical signals of SIFY remained relatively stable for 300 s at 200 °C, indicating the continuous operational performance of SIFY under high temperatures (Figure S9, Supporting Information).

### 2.4 Triboelectric Properties of SIFY E‐Textile

**Figure 4:**
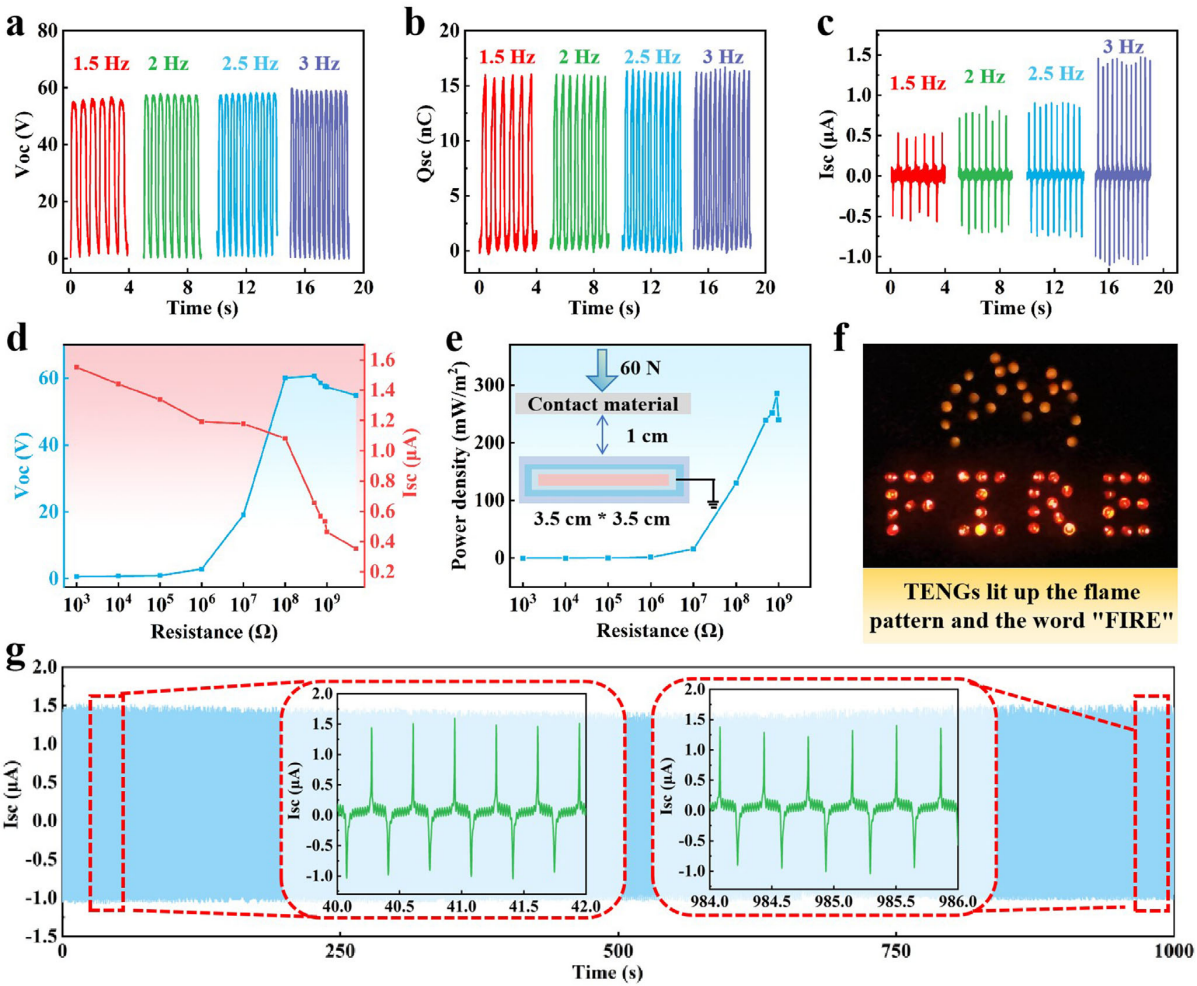
Electrical properties of SIFY e‐textile. a–c) V_OC_, Q_SC,_ and I_SC_ of SIFY e‐textile. d,e) Relationships between the V_OC_, I_SC,_ and power density of SIFY e‐textile and the load resistance. f) SIFY e‐textile lighting up 57 LEDs. g) The electrical performance of the SIFY e‐textile during 1000 s continuous operation at a frequency of 3 Hz.

SIFY boasts enough strength to be knitted into fabric‐based TENGs on commercial weft‐knitting machines. **Figure** **4**a–c illustrates the electrical output performance of SIFY e‐textile when fully contacted and separated from PU under a 60 N impact force at frequencies ranging from 1.5 to 3 Hz. As the impact frequency increases, V_OC_ and Q_SC_ of the SIFY e‐textile remain at 58.8 V and 16.4 nC, respectively, while the I_SC_ rises from 0.5 to 1.4 µA. Moreover, by connecting external resistors (ranging from 1 KΩ to 1000 MΩ), the electrical output of the SIFY e‐textile under 3 Hz excitation was investigated. As shown in Figure 4d, when the external resistance increases from 1 KΩ to 1000 MΩ, the V_OC_ rises from 0.61 to 57.3 V, and the I_SC_ drops from 1.55 to 0.46 µA. By connecting different resistors, the power density of SIFY e‐textile was studied. When the load resistance is 900 MΩ, the power density reaches 285.6 mW m^−2^ (Figure 4e). The SIFY e‐textile can serve as a power source. When the 3.5 cm × 3.5 cm SIFY e‐textile is tapped by hand, an LED array in the shape of “FIRE” and a flame logo, composed of 57 series‐connected LEDs, is lit up (Figure 4f; Video S3, Supporting Information). Moreover, the electrical energy generated by the SIFY e‐textile can be stored in capacitors. It can charge capacitors with capacitances of 2.2, 4.7, 6.8, and 10.0 µF within 300 s, and the charging rate decreases with the increase of capacitance (Figure S10, Supporting Information). To further demonstrate the electrical stability of the SIFY e‐textile, its electrical properties were continuously tested for 1000 s at a frequency of 3 Hz. Throughout the whole test, I_SC_ scarcely exhibited any significant decline, indicating its good stability (as shown in Figure 4g).

### 2.5 Durability of SIFY and Its E‐Textile

**Figure 5:**
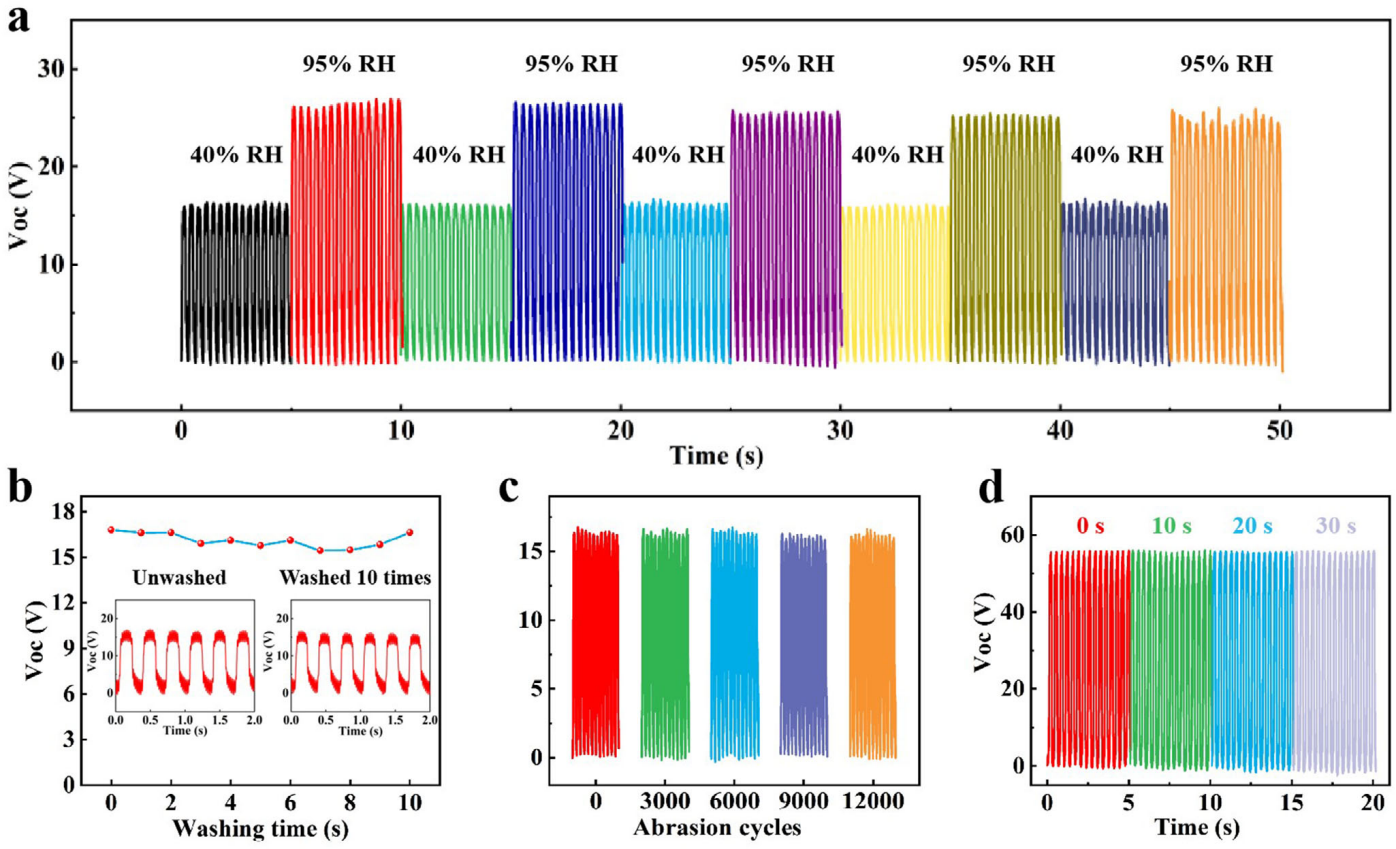
Durability performance of SIFY and its e‐textile. a) The output of SIFY in alternating dry (40% RH) and wet (95% RH) environments. b) Washability of SIFY. c) Wear resistance of SIFY. d) The V_OC_ of the SIFY e‐textile when it is subjected to an alcohol‐lamp flame for varying durations.

The stability and durability of SIFY and its textiles under the usage environment are crucial factors determining whether they can be applied in practice. When applied in firefighting suits, they inevitably encounter the harsh test of high‐humidity environments. Triboelectric e‐textiles are extremely sensitive to humidity. Generally, an increase in humidity often leads to a significant decline in the triboelectric output performance. In view of this, we conducted an in‐depth study on the electrical output performance of SIFY under high and low‐humidity cycle conditions. During the humidity increasing from 40% to 95%, the V_OC_ of SIFY rose from 16.7 to 25 V, as shown in **Figure** **5**a. When the humidity dropped back to 40%, its output could return to the initial level. After multiple humidity cycle tests, the electrical output of SIFY remained stable under high‐humidity conditions.

Wash resistance and abrasion resistance are essential properties for apparel fabrics. Notably, the V_OC_ of SIFY shows no significant change even after being washed 10 times (Figure 5b), demonstrating its excellent wash resistance. A single‐yarn abrasion tester (Figure S11, Supporting Information) was employed to document the condition of SIFY during different abrasion cycles. After 12 000 abrasion cycles, the structure of SIFY remained intact (Figure S12, Supporting Information). As depicted in Figure 5c, the V_OC_ of the yarn scarcely changed after 12 000 abrasions, stably maintaining at 16–17 V. This indicates that the SIFY possesses excellent wear resistance.

When SIFY is utilized for firefighting suits, its high‐temperature resistance and stability under the intense scorching of flames are of critical significance. To validate the application of SIFY e‐textile in fire protection, we fabricated fabrics from SIFY and carried out a series of tests. Figure 5d depicts the V_OC_ of the SIFY e‐textile when it is subjected to an alcohol‐lamp flame approximately for varying durations. After the fabric is exposed to the flame for 30 s, there is no apparent combustion damage on its surface, and only the internal SEBS/APP/PNFR experiences expansion of SIFY (Figure S13 and Video S4, Supporting Information). Additionally, the V_OC_ of the fabric scarcely changes after being burned for 30 s, which attests to its outstanding flame‐retardant property.

### 2.6 Applications of SIFY and its E‐Textile

Currently, commercial sensors can be applied in smart fire suits to assist firefighters in rescue operations.^[^ ^29^ ^]^ However, these sensors rely on batteries for power. In complex and ever‐changing fire scenarios, batteries are prone to damage or even explosion, which may pose new safety risks to firefighters.^[^ ^30^, ^31^, ^32^ ^]^ Meanwhile, during fire rescue operations, voice commands may be masked by the noisy environment, and thick smoke can also block visual signals, making it difficult for firefighters to communicate and convey instructions. The SIFY and its E‐Textile as a triboelectric nanogenerator is multifunctional and has outstanding advantages. It can serve as both a power source for other sensors and a self‐powered sensor.^[^ ^33^, ^34^, ^35^ ^]^ Moreover, unlike ordinary batteries, it has no leakage risk, offering higher safety and reliability.^[^ ^36^ ^]^

**Figure 6:**
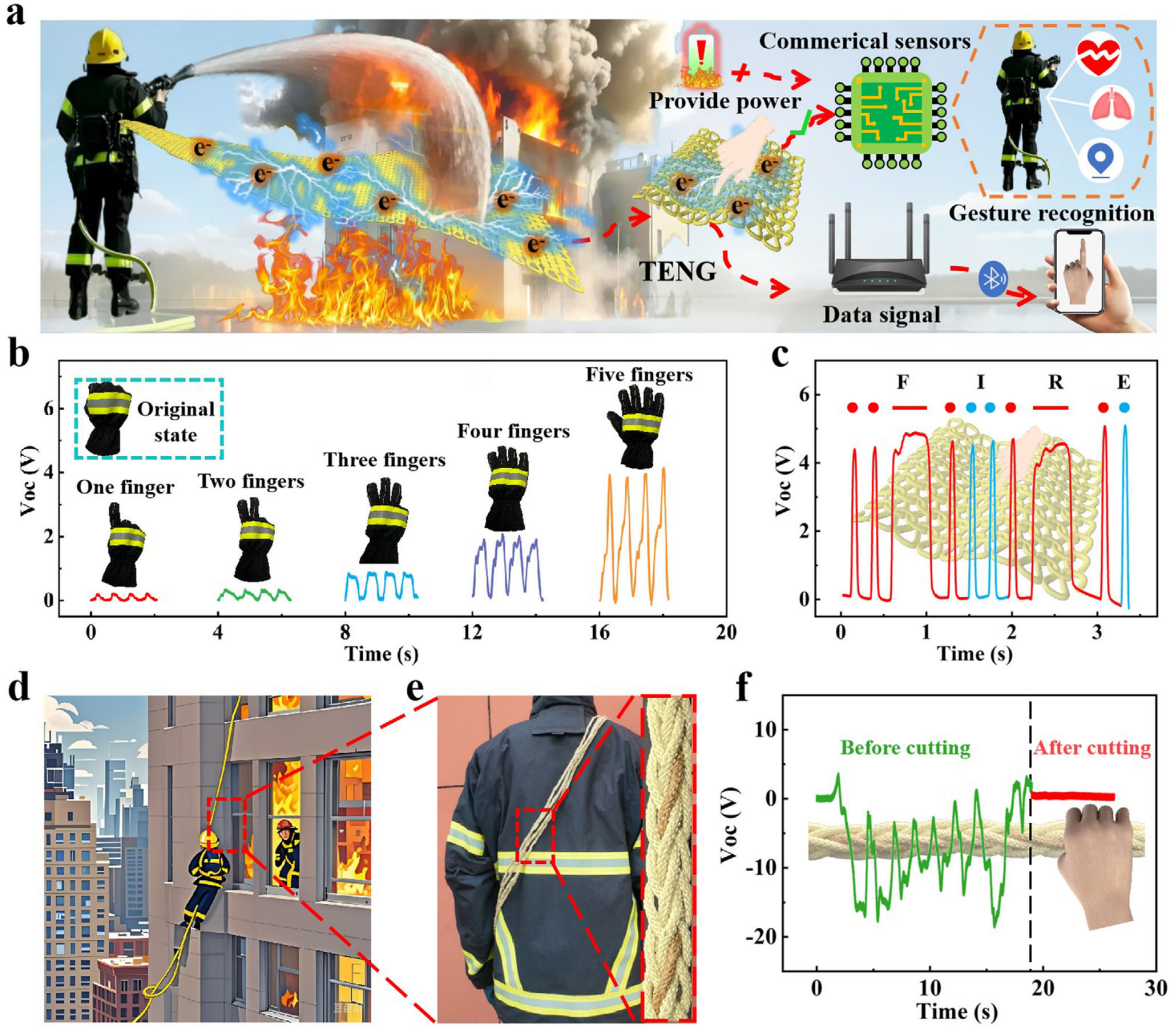
Applications of SIFY and its e‐textile. a) Schematic diagram of the application of SIFY e‐textile integrated into firefighting clothing. b) V_OC_ of SIFY for different hand gestures. c) V_OC_ corresponding to the Morse code “FIRE” when tapping on SIFY e‐textile. d) An application scenario diagram of the intelligent rope. e) A physical picture of the intelligent rope. f) The V_OC_ of the intelligent rope before and after damage.

**Figure** **6**a presents the application schematic of SIFY e‐textile integrated within the fire suit. When firefighters apply pressure to the SIFY e‐textile, it generates electrical energy, which can be harnessed to power low‐power‐consumption sensors. In addition, SIFY and its e‐textiles can function as sensors for gesture recognition and Morse code. The electrical signals produced by SIFY are precisely converted into corresponding gesture information by the data processor. Subsequently, this information is rapidly transmitted to the firefighters' mobile terminals through wireless transmission technologies such as Bluetooth, thereby assisting firefighters in clearly understanding instructions. Figure 6b depicts the output voltage signals of SIFY for different hand gestures. As the number of fingers in contact with the SIFY e‐textile increases from 1 (yielding an output voltage of ≈0.23 V) to 2 (≈0.36 V), 3 (roughly 0.89 V), 4 (≈2.01 V), and 5 (≈3.93 V), the V_OC_ of SIFY exhibits a substantial increase. Evidently, the real‐time V_OC_ can represent different hand gestures. Additionally, when fingers press the SIFY e‐textile for varying durations, different signals are generated. This characteristic enables the application of SIFY in Morse code compilation. As illustrated in Figure 6c, when fingers tap out the Morse code for a character, such as “FIRE”, the SIFY e‐textile generates corresponding electrical signals in real‐time. These signals can then be decoded by an appropriate decoder. Beyond electrical performance, response time constitutes a critical parameter. Experimental results show that the response time of SIFY in gesture recognition varies with the number of fingers in contact with the SIFY electronic textiles: increasing from 1 finger (210 ms) to 2 fingers (228 ms), 3 fingers (290 ms), 4 fingers (370 ms), and 5 fingers (371 ms). Furthermore, the response times of SIFY in Morse code (e.g., for short signals and long signals) are 54 ms and 183 ms, respectively. These response times effectively meet the interaction requirements (Figure S14, Supporting Information).

During fire rescue operations, firefighters frequently rely on fire ropes to aid in their work.^[^ ^37^ ^]^ However, if these ropes become worn or break without being noticed, firefighters are placed in mortal danger, particularly during high‐altitude rescues (Figure 6d). To solve this problem, we combined SIFY with aramid yarn to braid intelligent ropes (Figure 6e). These ropes are equipped with real‐time monitoring functions, which can promptly report the condition of the ropes and issue early warnings, greatly enhancing the safety protection for firefighters. During climbing, when the hand contacts and releases the ropes, friction occurs, generating electrical signals. The condition of the ropes can be judged by these electrical signals (Figure 6f). When the ropes are in good condition, electrical signals are output when firefighters grip and climb with them (Video S5, Supporting Information). When the SIFY yarn in the rope is damaged by abrasion, no electrical signal will be output (Video S6, Supporting Information). Based on the status of the electrical signals, the fire department can detect potential risks of the ropes in advance, issue early warnings, reduce risks, and firmly establish a safety line for fire rescue operations.

## 3 Conclusion

In summary, we successfully developed a mechanically robust, flame‐retardant, and humidity‐enhanced yarn‐based TENG by wet spinning and two‐dimensional braiding, as well as demonstrated its application in intelligent fire protection. Due to the existence of the SEBS/APP/PNFR and PBO fibers, the SIFY has excellent flame retardancy (LOI > 50%), mechanical properties (tensile strength of 550.72 MPa), and wear resistance (more than 12 000 times). Moreover, the triboelectric performance of SIFY increases with the increase of humidity, which breaks through the limitation of triboelectric nanogenerators in high‐humidity environments. Meanwhile, the SIFY e‐textile maintained stable V_OC_ after being exposed to the flame for 30 s. Based on the excellent flame retardancy, mechanical properties, the SIFY can be applied in gesture recognition technology and is capable of accurately capturing the subtle changes in electrical signals generated by hand movements. The intelligent fire‐fighting rope can determine its safety based on the presence or absence of electrical signals, providing a solid guarantee for the life safety of firefighters. At the same time, the flame‐retardant and waterproof properties enable SIFY to function normally in harsh environments such as high‐temperature and humid conditions. Under the laboratory conditions, it meets extreme firefighting requirements and can effectively enhance the safety and efficiency of rescue operations, while the field testing before pilot‐scale upscaling should be need to be considered. Overall, we anticipate that SIFY and its e‐textile will serve as an excellent candidate selection for the gesture‐recognition systems and intelligent fire‐fighting ropes, and thus contribute to the development of intelligent firefighting supplies.

## 4 Experimental Section

### Materials

The silver‐plated copper wire with a diameter of 0.08 mm was purchased from Qinghe Aoshuo Metal Material Co., Ltd. (Hebei, China). The PDMS solution consists of PDMS prepolymer and curing agent (Sylgard 184, Dow Chemical Company, USA). SEBS was purchased from Dongguan Xinmiao New Material Co., Ltd. APP was purchased from Aladdin (Shanghai, China). PNFR was purchased from Guangzhou Yinyuan New Material Co., Ltd. (Guangzhou, China). PBO fibers were purchased from Zhongke Jinqi New Material Technology Co., Ltd.

### Preparation of Stretchable Conductive Yarn

Preparation of PDMS Fibers: First, mix the PDMS prepolymer and the curing agent at a mass ratio of 10:1 at room temperature to form the spinning solution. Then, extrude the spinning solution through spinning holes into a 180–230 °C high‐temperature oil bath to form pre‐fabricated PDMS fibers. Finally, immerse them in diluted absolute ethanol for 30 min to remove surface residual oil and get the final PDMS fibers.

### Preparation of Stretchable Conductive Yarn

With the PDMS fiber serving as the core yarn and the silver‐plated copper wire as the winding yarn, the silver‐plated copper wire is uniformly wound around the PDMS fiber at a fixed angle. This process enables the fabrication of the stretchable conductive yarn, namely PDMS/Silver‐plated copper wire.

### Preparation of FWY

Preparation of SEBS/APP/PNFR solution: Disperse APP and PNFR in a specific ratio in a tetrahydrofuran solution, add an appropriate amount of SEBS particles, and stir the mixture at room temperature for 12 h.

### Preparation of FWY

FWY is obtained by coating the SEBS/APP/PNFR solution onto the PDMS/silver‐plated copper wire using coaxial wet spinning technology.

### Preparation of SIFY

Using the FWY as the core yarn and twelve PBO fibers as the outer wrapping yarns, SIFY was successfully fabricated through coaxial two‐dimensional braiding technology (Figure S15, Supporting Information).

### Fabrication of SIFY E‐Textile

The SIFY E‐Textile was knittted by SIFY on a weft knitting machine purchased from Taiwan Shengmei Machinery Co., Ltd. (Figure S16, Supporting Information).

### Characterizations

The surface and cross‐sectional morphologies of SIFY were observed through a super‐field microscope (VHX‐5000, China) and a scanning electron microscope (SEM, Quanta‐450‐FEG+X‐MAX50, FEI, Switzerland). The infrared spectra and the XPS spectra of the samples were characterized by the FTIR spectrometer (Nicolet iS20, USA) and XPS equipment (Thermo Scientific K‐Alpha, USA), respectively. The mechanical properties were tested using a universal testing machine (UTM5205X, China) in accordance with the GB/T 1040 standard. The electrical output was recorded by a Keithley 6514 system electrometer from Tektronix. The excitation power and frequency were determined by a vibration generator (JZK‐10, Guangdong, China). The force signal was monitored by a miniature weighing gravity sensor (ZN5S‐F, Anhui, China). The limiting oxygen index (LOI) value of the SIFY e‐textile was determined using an oxygen index tester (AUTO–YZS, manufactured in China) in accordance with the GB/T 2406.2–2009 standard. The physical object combustion experiment was carried out in an air atmosphere. A flame with a length of ≈1–2 cm was applied to the sample, which could burn continuously for 12 s to test its flame‐retardant performance. In compliance with the international standard EN388, the cut resistance performance of the fabric was measured using a cut‐resistant glove testing machine (QC‐200A, China). In accordance with the standard of ZBW 04005–89, the abrasion resistance of the yarn was tested using a computerized yarn abrasion tester (LFY‐109B, China). The SIFY was washed in a rigorous laundering environment according to GB/T 3921‐2008.

## Conflict of Interest

The authors declare no conflict of interest.

## Supporting information



Supporting Information

Supplemental Video 1

Supplemental Video 2

Supplemental Video 3

Supplemental Video 4

Supplemental Video 5

Supplemental Video 6

## Data Availability

The data that support the findings of this study are available from the corresponding author upon reasonable request.
