# Supplementary material for: Hierarchically Engineered Flame‐Retardant Triboelectric Yarn Exhibiting Robust Mechanical Performance and Humidity‐Boosted Electrical Output for Firefighting Applications
Source: Adv Sci (Weinh). 2025 Aug 12;12(41):e07673. doi: 10.1002/advs.202507673 (PMC12591168; doi:10.1002/advs.202507673)
Supplement: Supplementary file 1 — Supporting Information [file ADVS-12-e07673-s003.docx]

Supporting Information

**Hierarchically Engineered Flame-Retardant Triboelectric Yarn Exhibiting Robust Mechanical Performance and Humidity-Boosted Electrical Output for Firefighting Applications**

*Ying Sun ^a,1^, Yong Zhang ^a,1^, Haoyu Shi ^a^, Jinlin Liu ^a^, Yu Luo ^a^, Cheng Zhang ^a^, Kun Zhang ^b^, Shi-xiong Li ^c^, Shujuan Wang ^d,^*, Wei Fan ^a,^**

^a^ School of Textile Science and Engineering, Key Laboratory of Functional Textile Material and Product of the Ministry of Education, Xi’an Polytechnic University, Xi’an, Shaanxi 710048, China

^b^ College of Textiles, Donghua University, Shanghai 201620, China

^c^ Shaanxi Yuanfeng Prosafe Co., Ltd., Xi’an, Shaanxi 710025, China

^d^ School of Chemistry, Xi’an Jiaotong University, Xi’an, Shaanxi 710049, China

*E-mail: fanwei@xpu.edu.cn (Wei Fan); shujuanwang@mail.xjtu.edu.cn (Shujuan Wang)

1. **Supporting Figures**


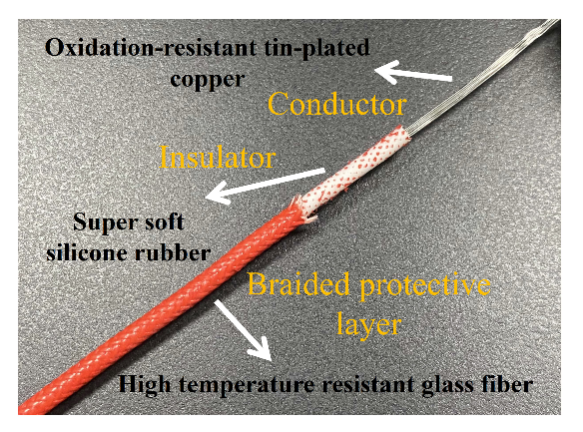


**Figure S1.** The structure of high-temperature resistant wire.


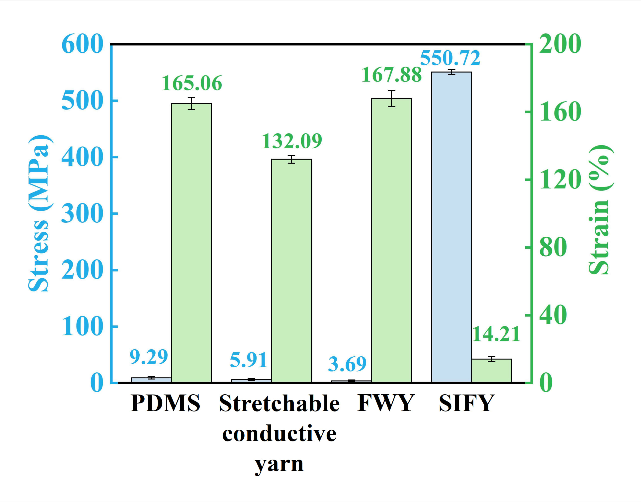


**Figure S2.** The mechanical properties of each part of SIFY.


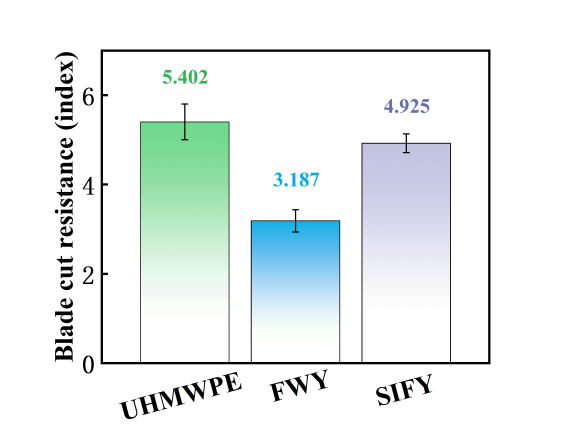


**Figure S3.** The cut resistance indices of ultra-high molecular weight polyethylene (UHMWPE), FWY and SIFY fabrics


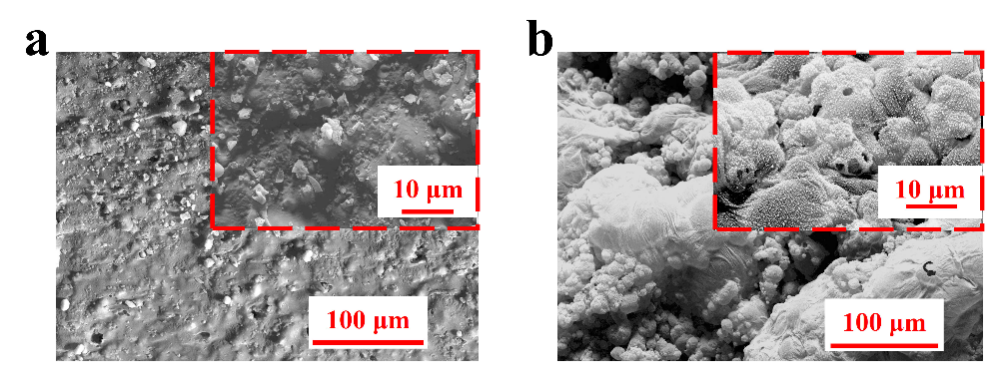


**Figure S4.** The SEM images of SEBS/APP/PNFR before and after the LOI test. a) Before the LOI test. b) After the LOI test.


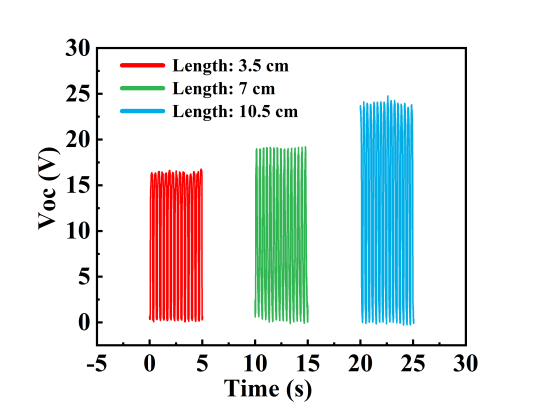


**Figure S5.** The output voltage of SIFY yarns with different lengths.


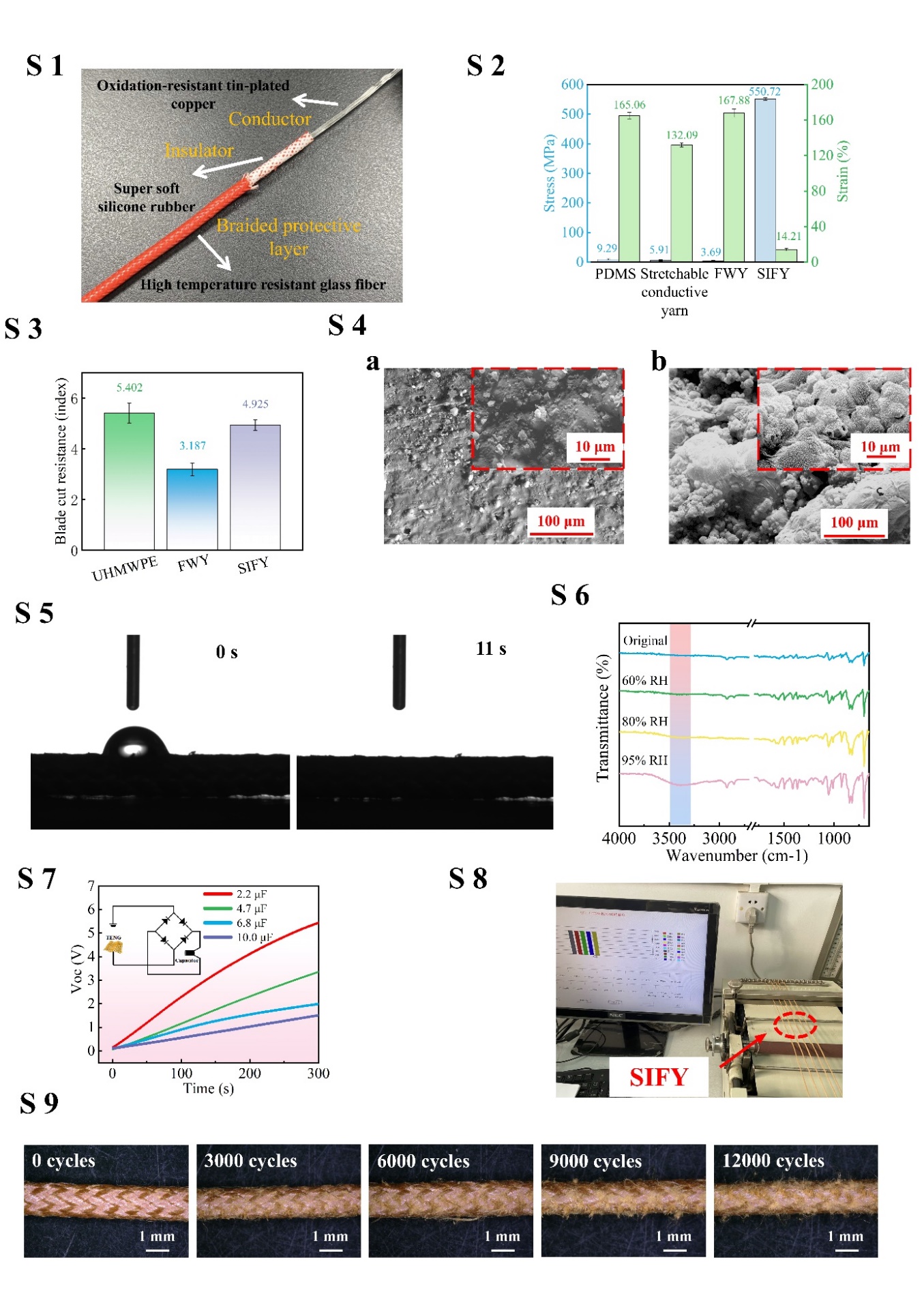


**Figure S6.** The states of water droplets on the surface of SIFY at different times.


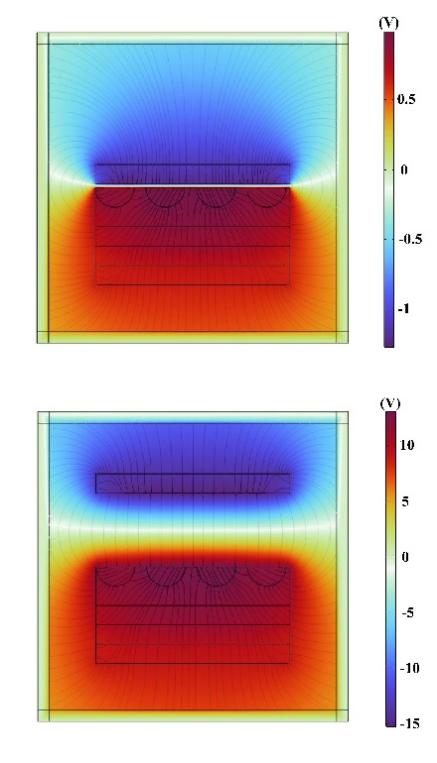


**Figure S7.** The electrostatic potential distribution of SIFY with moisture involvement obtained from COMSOL simulations


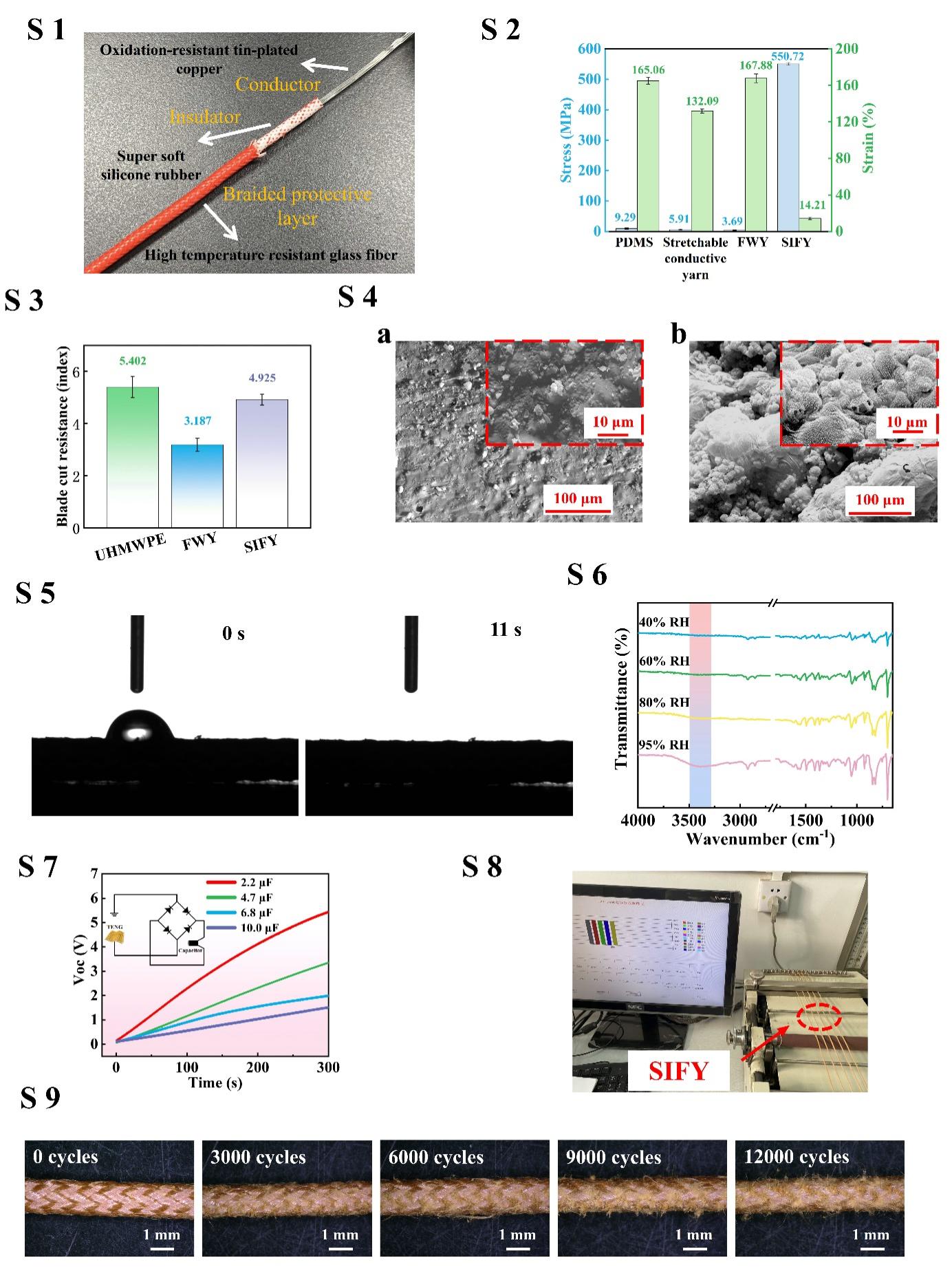


**Figure S8.** Infrared spectra of PBO fibers under different humidity condition.


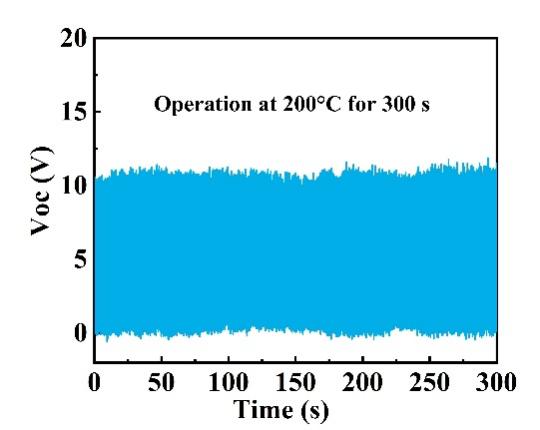


**Figure S9.** Electrical signal stability of SIFY during 300 s operational testing at 200°C.


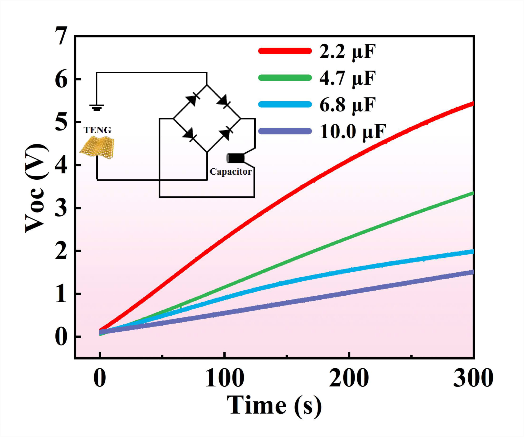


**Figure S10.** The V_OC_-time curve of SIFY e-textile for charging different capacitors (under 3 Hz, 60 N).


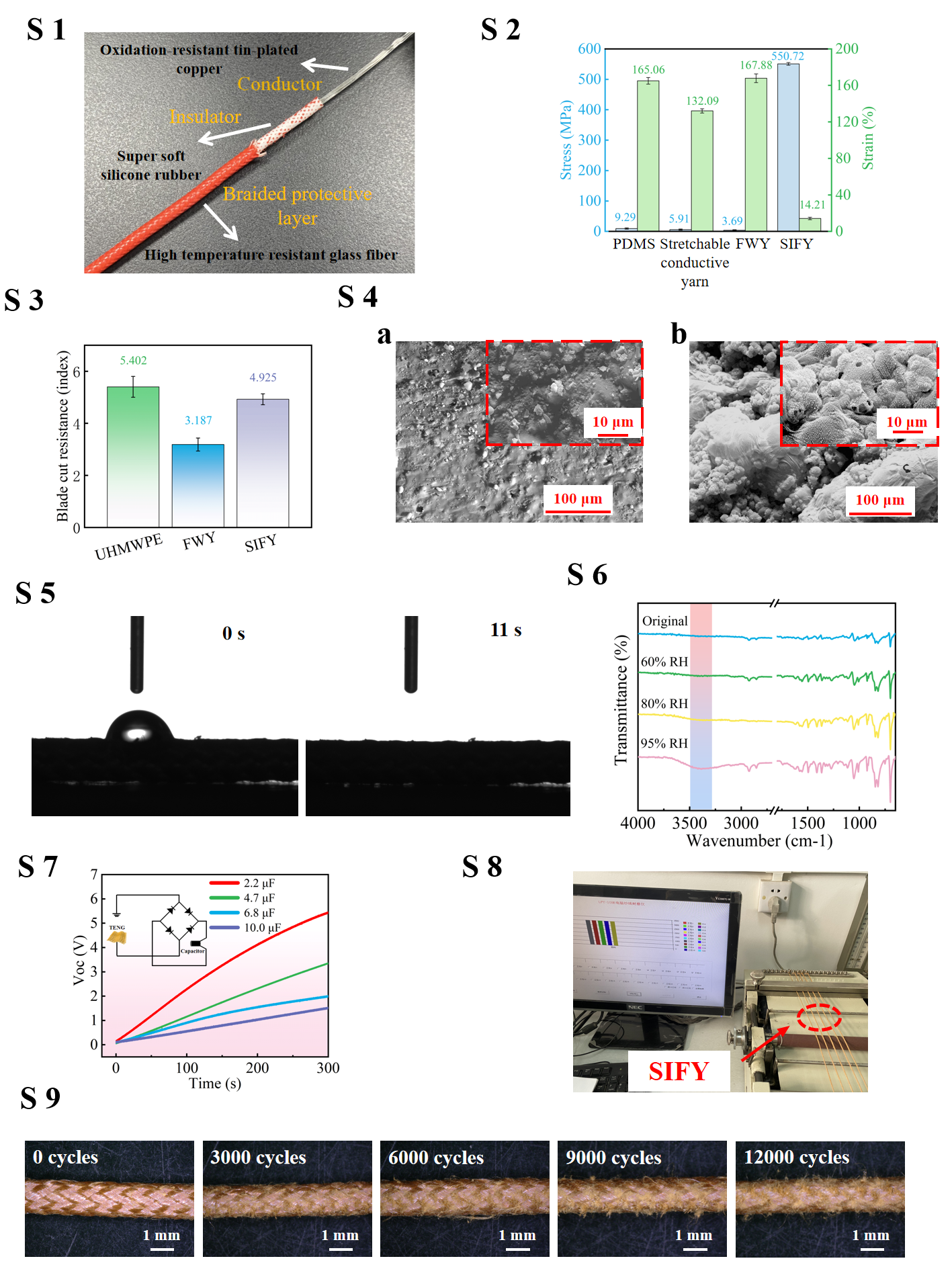


**Figure S11.** Physical image of the abrasion resistance test of SIFY.


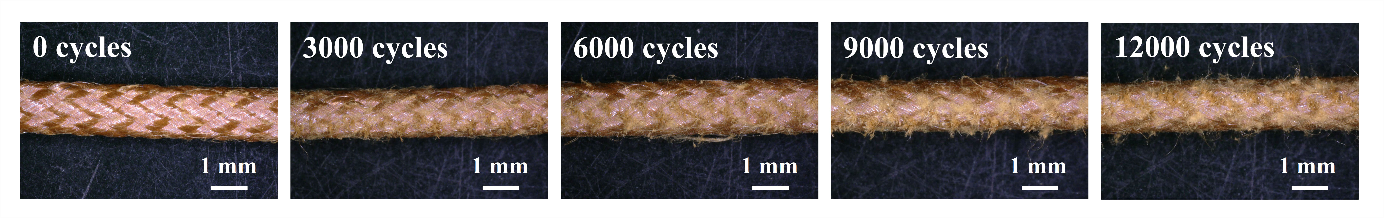


**Figure S12.** The surface morphology of SIFY under different wear cycle conditions.


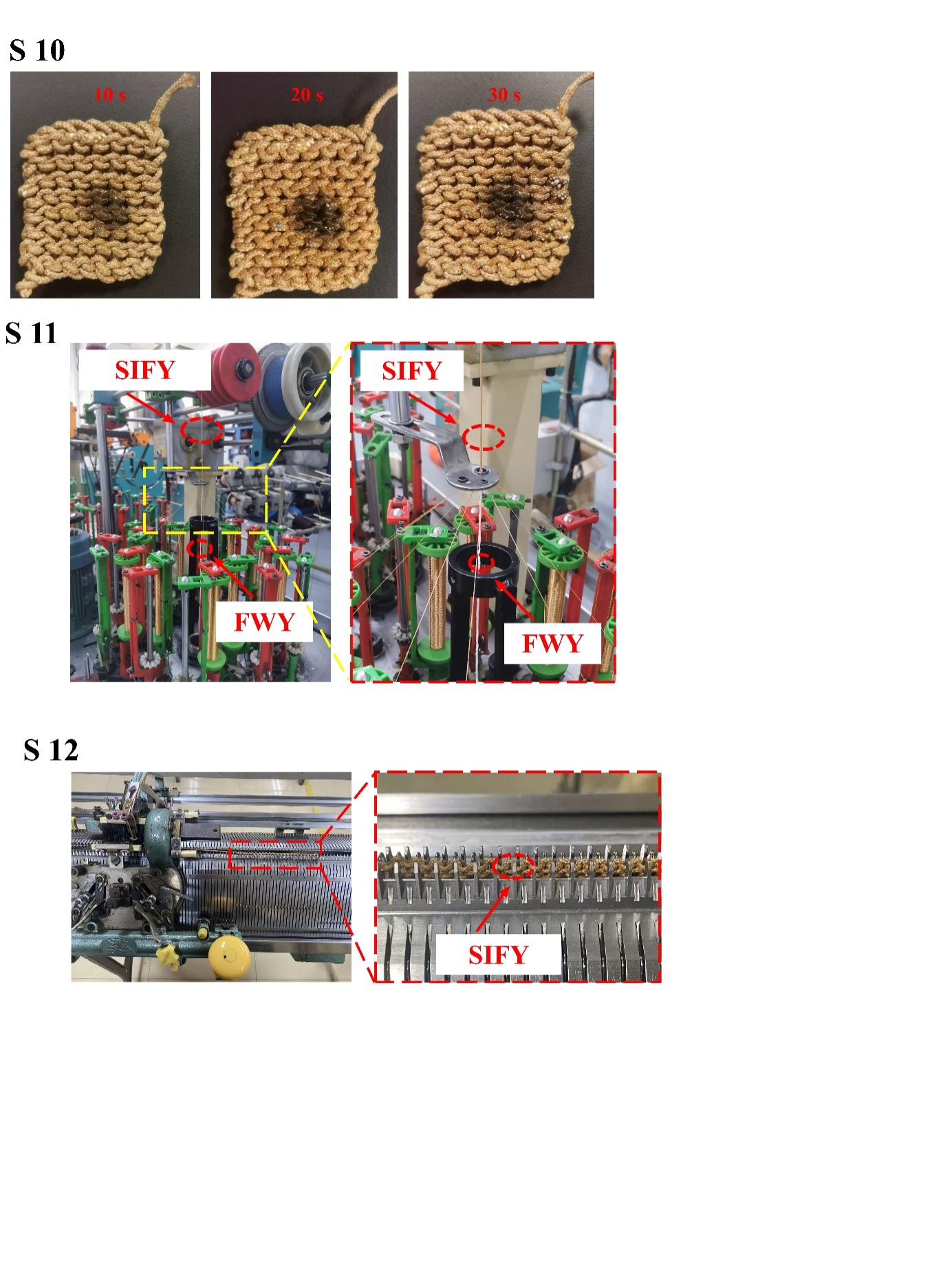


**Figure S13.** The physical pictures of the SIFY e-textile when it is subjected to an alcohol-lamp flame a for varying durations.


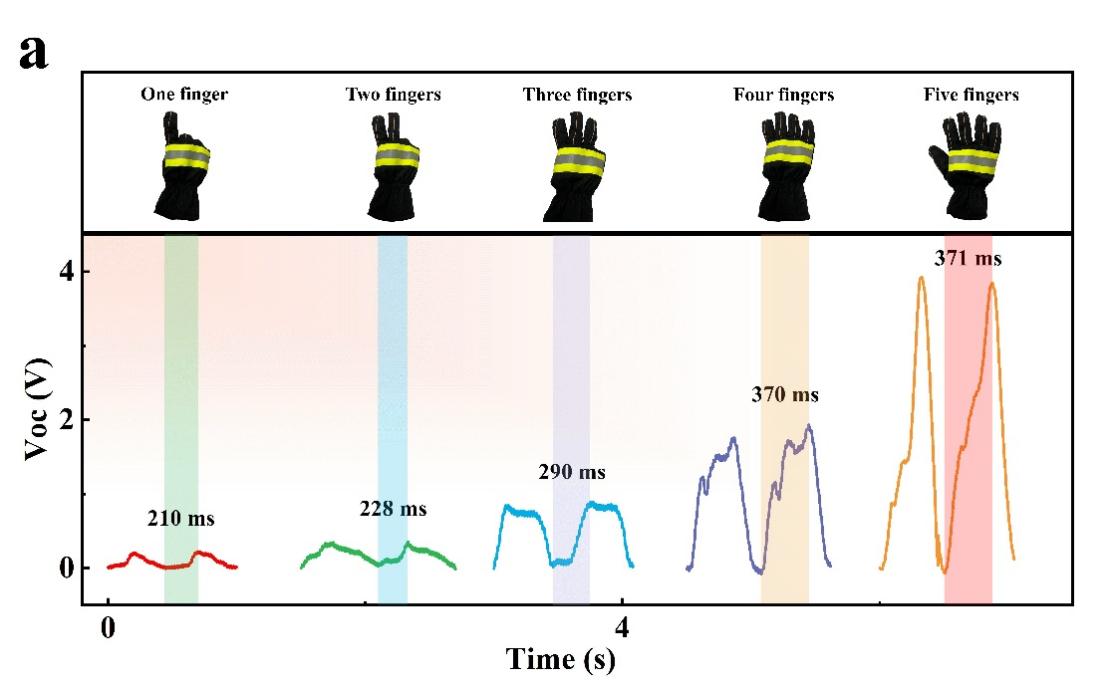

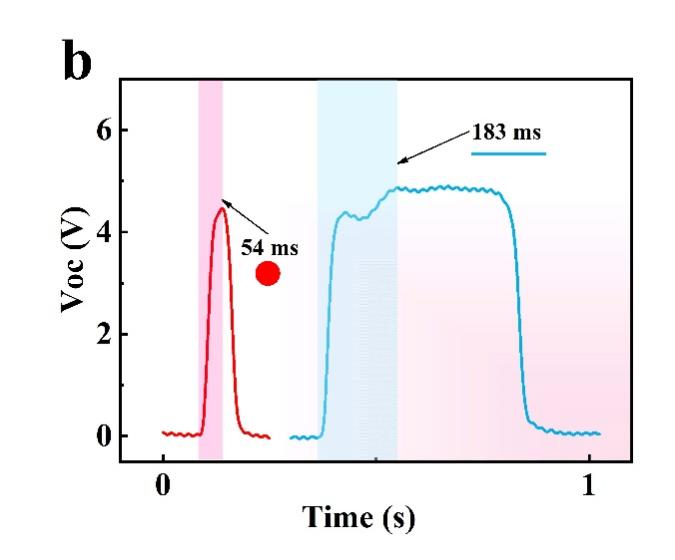


**Figure S14.** Response time of SIFY in both hand gesture recognition (a) and Morse code compilation (b).


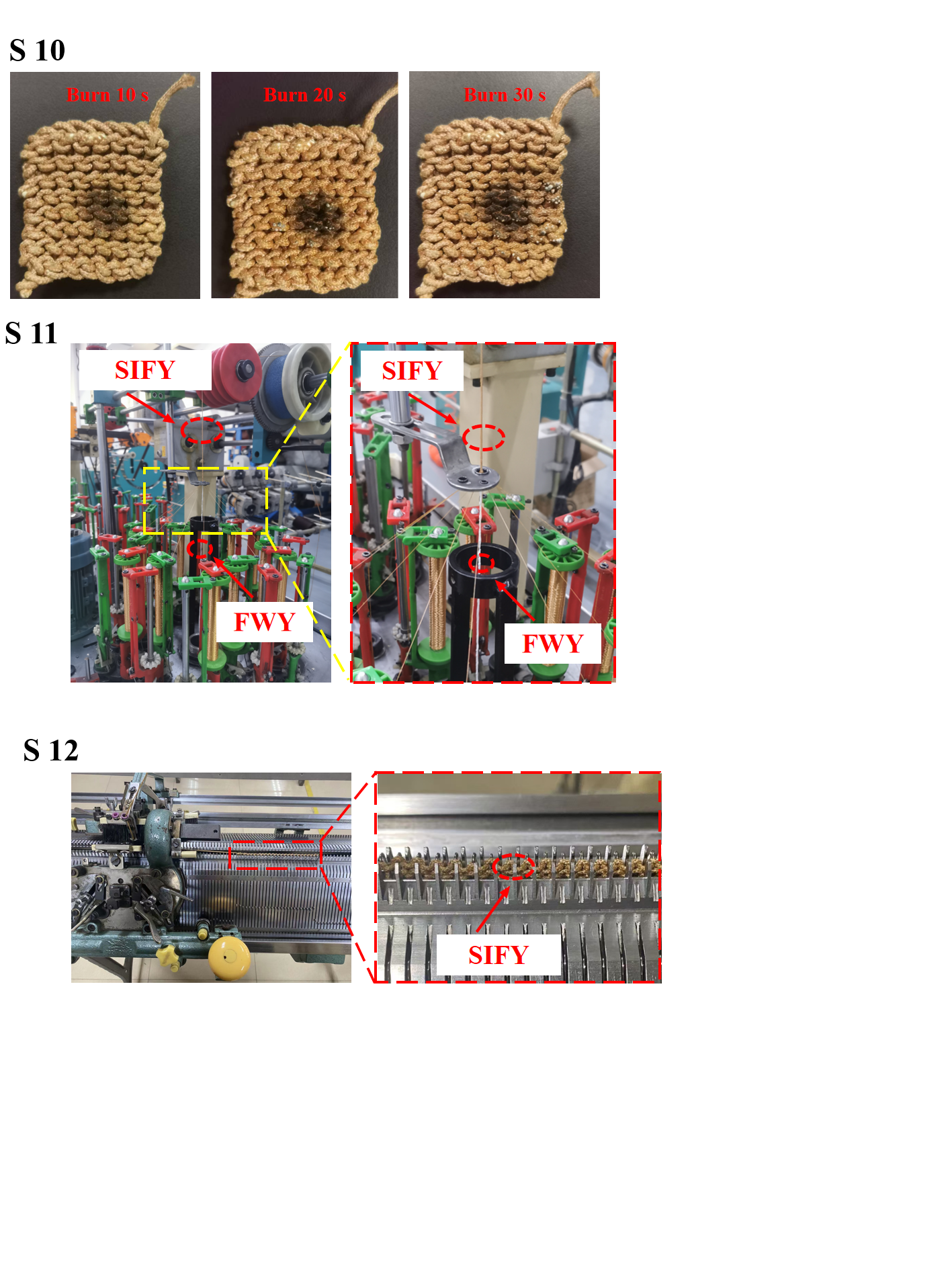


**Figure S15.** The two-dimensional braiding process for preparing SIFY.


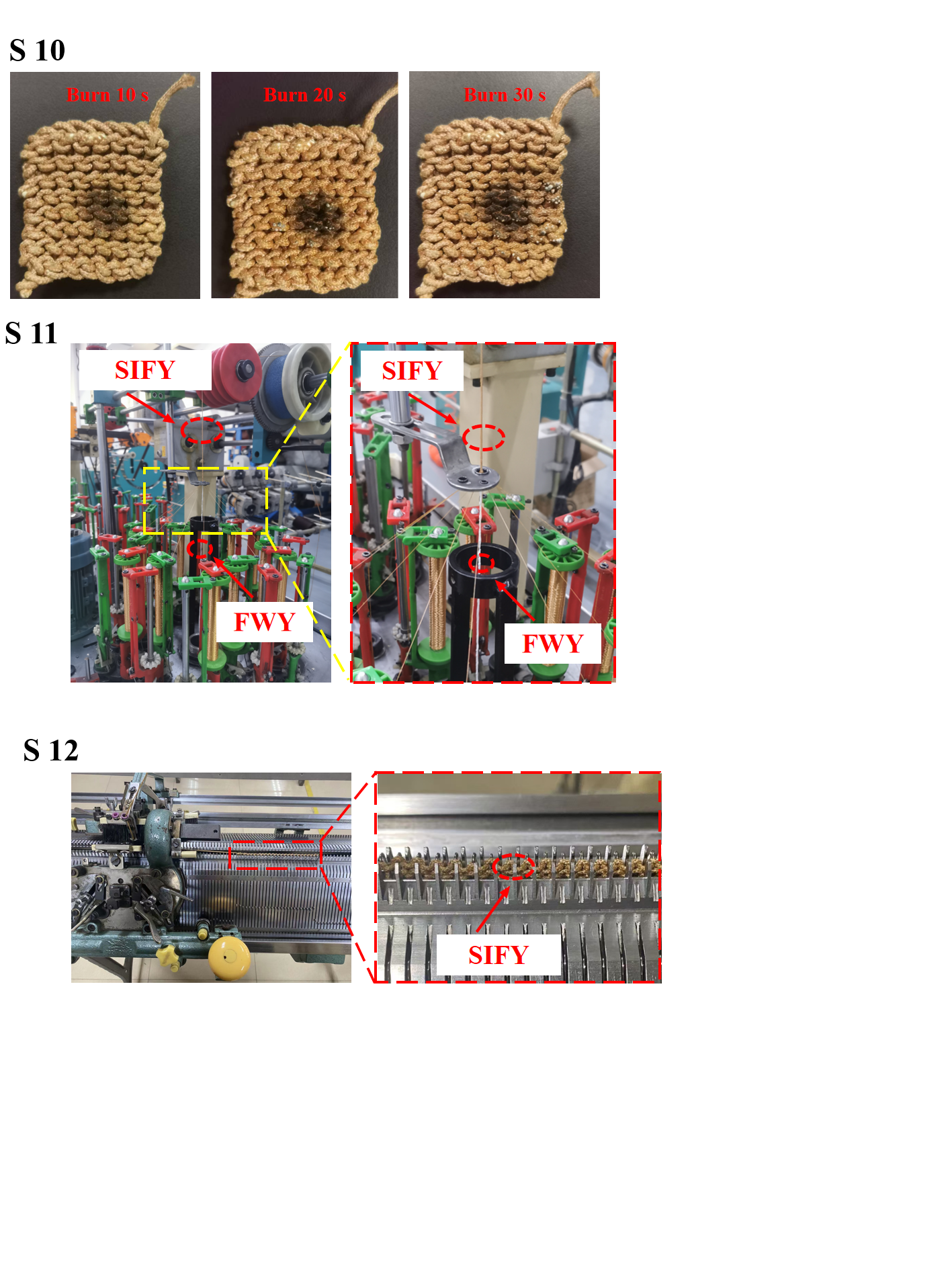


**Figure S16.** The process of weaving SIFY e-textile.

1. **Supporting Table:**

**Table** **S1**. Comparison of high-temperature electrical retention rate, humidity electrical retention rate, limiting oxygen index, strength and cut resistance between this work and the currently reported triboelectric yarns.

| Sample | High-temperature electrical retention rate | | Humidity electrical retention rate | Limiting oxygen index | Stress | cut resistance | Ref. |
| --- | --- | --- | --- | --- | --- | --- | --- |
| Fe_3_O_4_/Ag NWs/calcium alginate | 50.00% | / | | / | 13.1 MPa | / | [1] |
| Silver-plated nylon/FR-PET | / | / | | 31.3% | 218.0 MPa | / | [2] |
| Nickel-plated aramid/FPAA-FEP | 40.7% | 80% | | / | / | / | [3] |
| Pure copper yarn/PA11/ZnO/polyester staple | / | 36.0% | | / | 70.1 MPa | / | [4] |
| SIFY | 66.5% | 151.8% | | 50.2% | 550.7 MPa | 4.925 | This work |

1. **Supporting Videos:**

Video S1. Cut the fabric with a knife.

Video S2. Vertical burning process of the SIFY e-textile under an alcohol lamp flame.

Video S3. 57 LEDs lighted by the TENG fabric.

Video S4. The test process of SIFY e-textile Burning for 30s.

Video S5. The use of smart fire-fighting ropes (normal).

Video S6. The use of smart fire-fighting ropes (damaged).

**References:**

[1] H. He, J. Liu, Y. Wang, Y. Zhao, Y. Qin, Z. Zhu, Z. Yu, J. Wang, *ACS Nano.* **2022**, *16*, 2953-2967.

[2] X. J. Cui, A. Li, Z. N. Zheng, H. G. Wu, R. Wang, *Adv. Mater. Technol.* **2023**, *8*.

[3] M. Hao, X. Hu, Z. Chen, B. Yang, Y. Liu, Q. Wang, X. Gao, Y. Liu, X. Wang, Y. Liu, *ACS Appl. Mater. Interfaces.* **2025**, *17*, 1038-1048.

[4] W. C. Chen, W. Fan, Q. Wang, X. C. Yu, Y. Luo, W. T. Wang, R. X. Lei, Y. Li, *Nano Energy.* **2022**, *103*.
